# DF-4 Lead Connector: Innovative Technology, Unexpected Problems and Novel Solutions

**DOI:** 10.1016/s0972-6292(16)30751-3

**Published:** 2014-05-25

**Authors:** Kartikeya Bhargava

**Affiliations:** Senior Consultant Cardiology, Medanta-The Medicity, Gurgaon, Delhi- NCR, Haryana, India

**Keywords:** DF4 adaptor/splitter, high DFT

## Introduction

Implantable cardioverter defibrillator (ICD) technology has evolved from the surgical thoracotomy based device systems in 1980 to only transvenous lead based systems in 1990s to subcutaneous ICD systems more recently. As far as transvenous lead based ICD systems are concerned, major advancements have occurred that include decrease in ICD device size and use of biphasic shock waveform. Many software and algorithmic developments have redefined optimization of defibrillation waveform, supraventricular-ventricular tachycardia discriminators, avoidance of unnecessary right ventricular pacing, delivery of anti-tachycardia pacing during charging etc.. However, not all of the technological advances accepted for clinical use have been trouble free. Use of newer ICD lead designs with reduced caliber resulted in unanticipated problems like lead fracture or insulation failure in some of them warranting their recall [1]. Another recent advancement in the lead design is the development of DF-4 technology. The traditional DF-1 lead design has bi/trifurcated lead connectors that integrate in to a single lead body through a yoke. Use of the newer DF-4 ICD lead technology has simplified the implant process to some extent and has certain other advantages.

## DF-4 Design: Innovative Technology

 The DF-1 lead consists of a bifurcated (in a single coil lead) or trifurcated (in dual-coil lead) header connector pins - one pace-sense IS-1 connector and one or two DF-1 high voltage connectors. These connectors join together in to a yoke that then integrates these in to a single lead body, the distal end of which is implanted in the right ventricle. Thus, the device header has three plugs (single chamber ICD with dual-coil lead), four plugs (dual-chamber ICD with dual coil lead) or five plugs (CRT-D device with dual-coil ICD lead). This large header size along with yoke and its bi/trifurcation of the ICD lead makes the whole system quite bulky. There is small but definite risk of inadvertent connection of right ventricular coil plug into superior vena cava (SVC) coil port of the device header while using a dual-coil ICD lead. In addition, during device replacements, it becomes essential to free all components of the lead connector from the adhesions formed in the device pocket resulting in increase in risk of injury to the leads and prolonged procedure. Hence, there was a need to simplify the lead design such that only one connection is needed between the lead and the device.

The DF-4 standard was formalized in 2010 as a four-pole inline connector system and was endorsed by the Association for the Advancement of Medical Instrumentation in August 2011. [2] The standard allows interchangeability from all manufactures and ensures compatibility with future implanted devices. The DF-4 connector port cavity in the device provides a combined cavity with four contacts - two low-voltage for pace-sense and two high-voltage for defibrillation coils. A single distal set screw to the tip electrode holds the lead securely in the device. The DF-4 lead has only one plug providing four poles and does not have any bi/trifurcation or a yoke. The seals insulating the contacts are mounted in the device header and hence get replaced every time the device is replaced. In contrast the seal rings are part of the lead in the DF-1 design of the lead and are susceptible to wear and tear.

The actual or potential advantages of the DF-4 lead design over traditional DF-1 design include [3]:

1. Easier implantation with shorter procedure time.

2. Reduction of chances of pin-port misconnection due to reduced lead - device connections.

3. Smaller device due to reduction in header size.

4. Smaller and less bulky lead at the proximal side.

5. Increase device reliability due to lesser risk of lead to can abrasion.

However, all these potential advantages are more of a convenience to the implanter rather than obvious clinical necessity. Only, the long-term clinical use of these device systems will indicate whether these potential advantages have actual clinical benefit.

## DF-4 Design: Unexpected Problems

The DF-4 ICD lead may make the implant procedure simpler, but may pose known or unexpected problems. This system will not allow addition of a dedicated pace-sense lead in the right ventricle in case there is isolated failure or malfunction of pace-sense component of the ICD system. This is sometimes needed if issues like delayed increase in pacing threshold or marked reduction in sensed R waves or oversensing due to diaphragmatic potentials develop that cannot be tackled by appropriate reprogramming. In the DF-1 ICD system, a new additional right ventricular pace-sense lead can be implanted and its pin connected to the IS-1 port of the header of the device. The redundant IS-1 port of the ICD lead can be capped and secured in the pocket. However, the DF-4 system will need an entirely new ICD lead to be implanted to overcome this issue - the old lead then needs to be either extracted (preferably) or abandoned.

Some patients during generator change may not need the ICD or CRT-D but may still need pacing support for bradycardia or resynchronization. The patient may refuse the ICD or may have gone out of the indication for ICD due to increase in left ventricular function. The downsizing of the ICD to a pacemaker and CRT-D to CRT-P can easily be done in the DF-1 system by connecting the IS-1 port of the ICD lead to the IS-1 port in the header of pacemaker or CRT-P. However, this is not possible in the DF-4 system and may require implantation of a new additional pacing lead.

Similarly, the DF-4 ICD lead and device system does not permit addition of a shocking coil or an array to tackle high defibrillation threshold (DFT) detected at implant or diagnosed later during clinical follow up. A shocking coil or array can easily be added to the SVC coil port of the header of the ICD with DF-1 lead. However, in an ICD system with DF-4 design, it may necessitate replacement of both the ICD lead and the device with a new lead and device with DF-1 design along with implantation of new shocking coil or array. This can make the procedure very cumbersome with many potential risks and complications. Cogert et al [4] reported an actual case wherein the issue of high DFT warranted removal of the lead and the CRT-D device with DF-4 design and replacement with the traditional DF-1 ICD lead, CRT-D device and additional azygous coil.

## DF-4 Design: Novel Solutions

 New technology, though mostly advantageous, sometimes leads to new challenges and then novel solutions are offered to bail out the unexpected problems. In this issue of the journal, Inbar S et al report a case wherein high DFT was managed by adding an extra defibrillation lead to the DF-4 design ICD device using a high voltage adaptor/splitter [5]. This specialized Y adaptor (Medtronic 5019 HV splitter) has a DF-4 connector at one end (connects to the device header) and two separate connections (one for the DF-4 ICD lead and other for additional coil with DF-1 connector) at the other end. The connection for the DF-4 lead excludes the SVC coil. Hence, this adaptor can be used with both single coil DF-4 lead as in the case report published in this issue [5] or with dual-coil DF-4 ICD as published in a similar report [6] earlier. Use of this specialized adaptor permitted use of the same DF-4 ICD lead and device with addition of an azygous coil to manage high DFT and avoided an otherwise complex revision procedure.

 Although, use of such adaptor/splitter may have its own issues and problems, it can certainly help in bailing out a difficult case of high DFT in a DF-4 ICD system.

 An adaptor that allows addition of a new pace-sense lead is still not available. It would require a design similar to this adaptor with the one end having DF-4 connector (for the device) and other having two connectors - one for DF-4 ICD lead (that excludes the pace-sense port) and another having IS-1 connector for additional pace-sense lead. The need for such adaptor exists with increasing use of DF-4 systems and surely will be made available by the device manufactures in the near future.

## Conclusions

 Use of an adaptor/splitter for connecting a lead with a connector standard different from that in the device or for adding additional leads has been in vogue for a long time in the history of cardiac rhythm management. The development of connector pins with new international standards as exemplified by DF-4 design will continue to raise the need for new adaptors to manage unexpected problems and challenges associated with lead and device connections.
